# Agent-based modeling of morphogenetic systems: Advantages and challenges

**DOI:** 10.1371/journal.pcbi.1006577

**Published:** 2019-03-28

**Authors:** Chad M. Glen, Melissa L. Kemp, Eberhard O. Voit

**Affiliations:** The Wallace H. Coulter Department of Biomedical Engineering, Georgia Institute of Technology and Emory University, Atlanta, Georgia, United States of America; National Research Council of Italy, ITALY

## Abstract

The complexity of morphogenesis poses a fundamental challenge to understanding the mechanisms governing the formation of biological patterns and structures. Over the past century, numerous processes have been identified as critically contributing to morphogenetic events, but the interplay between the various components and aspects of pattern formation have been much harder to grasp. The combination of traditional biology with mathematical and computational methods has had a profound effect on our current understanding of morphogenesis and led to significant insights and advancements in the field. In particular, the theoretical concepts of reaction–diffusion systems and positional information, proposed by Alan Turing and Lewis Wolpert, respectively, dramatically influenced our general view of morphogenesis, although typically in isolation from one another. In recent years, agent-based modeling has been emerging as a consolidation and implementation of the two theories within a single framework. Agent-based models (ABMs) are unique in their ability to integrate combinations of heterogeneous processes and investigate their respective dynamics, especially in the context of spatial phenomena. In this review, we highlight the benefits and technical challenges associated with ABMs as tools for examining morphogenetic events. These models display unparalleled flexibility for studying various morphogenetic phenomena at multiple levels and have the important advantage of informing future experimental work, including the targeted engineering of tissues and organs.

## Background and history

Morphogenesis is the complex chain of biological processes with which cellular populations self-organize, in a reproducible manner, into predetermined structures or patterns. It involves a multitude of mechanisms and systems and is governed by signal transduction across various spatial and temporal scales. In addition to evident outward patterning events, such as the formation of stripes on a tiger’s back or the regular spacing of hairs or feathers, morphogenesis encompasses all molecular processes that convert a fertilized egg cell into a blastula, then into an embryo with germ layers that have their unique roles, and ultimately into a functional organism. It has been known for some while that the numerous simultaneous events during this journey, such as the development of fingers out of a limb bud or the organization of neurons into functional networks in the brain, involve fundamental processes of cell migration and differentiation, but it is extraordinarily difficult to ascertain and characterize the molecular, mechanistic underpinnings guiding these processes and allowing the often complicated structures to form.

Notwithstanding these challenges, the fact that an extremely complex organism evolves out of a single cell or a seemingly homogeneous group of cells is very intriguing, and it is hardly surprising that the biological and chemical study of morphogenesis eventually coalesced with mathematical—and later computational—approaches that attempted to distill the essence of pattern formation out of the overall complex developmental process. Whereas the first mathematical approaches relied on simple diffusion gradients and biochemical reactions, the emergence of unprecedented computer power and its wide accessibility increasingly permitted more complicated and realistic simulation studies, which have culminated by now in sophisticated agent-based models (ABMs). These models are uniquely qualified for spatially and functionally representing the complexity of a system that is the collective result of a multiplicity of well-timed, fine-tuned cues. This review summarizes the development of morphogenetic models from relatively simple reaction–diffusion (RD) models to today’s complex ABMs and places particular emphasis on proliferation, migration, and differentiation as the main mechanisms for pattern formation.

The history of morphogenetic observations and investigations goes back a long time, but theory-based explanations were not proposed until the 20th century. A landmark was D’Arcy Thompson’s work *On Growth and Form* [1], in which he described similarities between mechanical and physical systems and the shapes of biological organisms. Because of severe limitations with respect to both theoretical analysis and experimental validation, his observations and calculations were purely hypothetical, as he freely acknowledged. Nevertheless, they marked the beginning of an illustrious scientific development. A decade later, Alan Turing proposed in his treatise *The Chemical Basis of Morphogenesis* [2] a mechanistic explanation that dominated the field for several decades. The core concept of this theoretical explanation was the by now widely accepted RD mechanism, in which, under the right conditions, a two-molecule reaction system is capable of producing periodic patterning through diffusion instability. Specifically, a fast-diffusing global inhibitor interacts with a slow-diffusing local activator, and their functional coupling can be shown to exhibit nonlinear reaction dynamics that can generate repetitive patterns, such as spots or stripes [3]. For instance, the inhibitor prevents features such as hair follicles from forming too close to each other, an important and widespread effect sometimes called lateral inhibition [4].

The RD patterns produced by the inhibitor and activator gradients can be considered chemical prepatterns that act as templates for future differentiation. Thus, the apparent initial homogeneity of an egg or cell cluster morphs into spatially distinct profiles of “invisible” high and low concentration regions, which later guide the implementation of cellular fate decisions and the emergence of visible shapes and forms. Importantly for the field of computational morphogenesis, Turing’s RD mechanism demonstrated that it is feasible to represent morphogenetic patterns using a simple, biochemically plausible system governed by simple mathematical rules. Alas, although the theory was conceptually convincing, it did not gain significant traction in the field for almost five decades.

In another landmark publication, two decades after Turing’s proposal, Lewis Wolpert introduced the conceptual framework of positional information (PI) as a mechanism of pattern formation during morphogenesis [5]. This theoretical framework was inspired by old observations during the morphogenesis of sea urchins, which Hans Driesch had made as early as 1891 [6]. According to the tenets of PI, a cell is able to determine its assigned fate from its position relative to other parts of the organism [7, 8]. The position, in turn, is characterized by the concentration of a morphogen. Thus, a cell senses its positional value by interpreting a morphogen concentration and makes a fate decision based on this local information. It is assumed that the cell interprets the position based on its genetic makeup and its developmental history, but a central claim of PI is that there is no prepattern in the embryo. A reasonable biological implementation of PI could be a morphogen source leading to a spatial morphogen gradient that gradually decreases with the distance from the source, thereby providing PI. The concept of PI is quite intuitive and was quickly accepted in the field, partly because experimental findings corroborated its existence [9]. For instance, numerous experiments, especially in the field of limb development, regeneration, and transplantation, clearly suggested that cells indeed possess characteristic information regarding their position, which may be acquired through dedicated regulatory programs involving genes such as *sonic hedgehog* (*SHH*), *hunchback*, and *Hox* (e.g., [10]).

In spite of the intuitive appeal of PI and intense research over several decades, it remains unclear even today how the necessary gradient is established, how the cell senses it, and how a cell correctly interprets it. Diffusion comes to mind, but diffusion processes are not particularly reliable, precise, or robust toward external perturbations, and one must wonder how interactions between morphogens and their environment would be realized in terms of effective molecular events. Furthermore, although sensing of a morphogen by a cell is easy to imagine in principle, the cells along a gradient would have to be able to distinguish very subtle concentration differentials [11]. As Wolpert [8] himself recently stated, “There is no good evidence for the quantitative aspects of any of the proposed gradients and details of how they are set up.” Thus, to summarize PI, many experiments have convincingly suggested that PI exists, but it is not clear how it is implemented in living organisms. Finally, one should note that PI and RD are not mutually exclusive but in fact complementary (e.g., [12]).

The advent of the digital computer and its eventual widespread availability drastically accelerated research on morphogenesis. Computational modeling permitted additional complexity to be considered with minimal effort and effectively decreased the limitations caused by mathematical tractability. In particular, the RD mechanism experienced burgeoning interest [13–15], and the Turing paradigm was successfully put to the test with sophisticated differential equation modeling and improved experimental techniques. Ultimately, research in recent years has substantiated the role of RD mechanisms alongside PI mechanisms during development [16–20], despite their slow start. In addition to the two prominent morphogen-based mechanisms, newer approaches to understanding morphogenesis with computational means have also taken mechanical, electrical, and environmental cues into account [21–25]. However, similar to the treatment of chemical mechanisms, both mechanical and electrical cues have typically been investigated independently. This is a clear shortcoming, as these factors often act simultaneously and presumably synergistically. Thus, in moving forward, it seems wise to pay attention to the multiscale nature of morphogenesis and to the integration of the diverse signals that control it.

## A brief description of mathematical and computational approaches to morphogenesis

### RD strategies

The original RD system proposed by Turing was composed of two partial differential equations (PDEs) describing the spatiotemporal dynamics of two compounds, *A* and *B*. In generalized format, these equations read

$$\frac{\partial A}{\partial t}=D_{A}\nabla^{2}A+F(A,B),$$

$$\frac{\partial B}{\partial t}=D_{B}\nabla^{2}B+G(A,B),$$

where the ∇-terms represent diffusion and F and G are the nonlinear functions representing the reaction kinetics. For this system to achieve diffusion-driven instability, the nonlinearity of the reactions is obligatory, and the diffusivities between the two species must be different. Various particular reaction formats have been analyzed as pattern generators using the RD paradigm. The first examples satisfying these requirements were proposed several decades ago, for instance, by Gierer and Meinhardt [26] and by Schnakenberg [27]. At the time of these studies, computers did not have the bandwidth or speed for realistic simulation studies, which mandated mathematical solution by hand, which quickly became very laborious, given the sensitivity of the RD mechanism to parameter values and initial conditions. An important “trick” toward solving the equations was the transformation, or scaling, of the equations into a nondimensional format. The resulting dimensionless equations maintain continuity between diffusion and reaction terms of both species but retain the feature of being quite general with respect to parameter values and also simplify the visualization of the admissible parameter space. Murray provided solutions for this set of nondimensional RD systems and describes a methodology to isolate the Turing space, which consists of the range of parameter values that successfully generate patterns [28].

**Fig 1:**
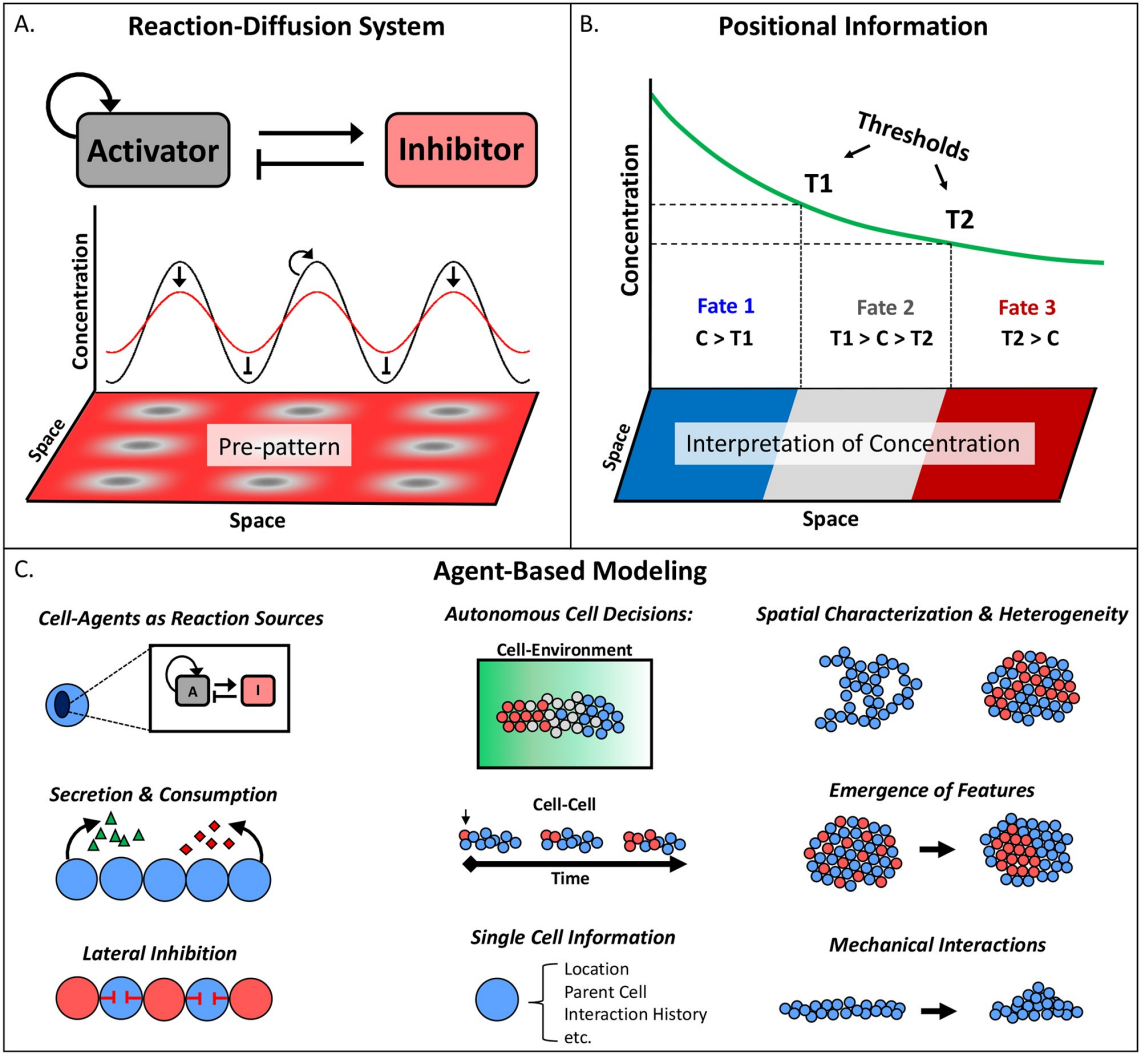
Overview of the two most popular theories for pattern formation during morphogenesis, reaction–diffusion systems and positional information, as well as common features of agent-based models for morphogenesis. (A) Archetypal Turing reaction–diffusion system with an activator and inhibitor generating repetitive patterns from differential diffusivities and nonlinear reaction terms. The reaction–diffusion system depends on the concept of a chemical prepattern developing in advance of cell fate decision and emphasizes the ability to induce pattern formation from an allegedly homogeneous initial state. (B) Wolpert’s positional information theory proposes an interpretation step based on concentration thresholds that alleviates the need for a morphogenetic pattern to match the chemical prepattern. In positional information, a cell is capable of multiple fate decisions from a single molecular gradient by discerning subtle variances in concentration along the gradient. (C) Agent-based modeling provides a framework capable of implementing features from both theories. Cell agents can act as the sources of activators (“A”) and inhibitors (“I”), permit localized reactions, and make autonomous decisions in response to their local environment. In addition to the generation of static patterns, agent-based modeling allows for the investigation of dynamic, spatiotemporal patterning.

Structurally, the RD approach has not changed much since its inception: it is still mostly driven by two interacting molecules and uses PDEs, inspired by continuum mechanics, on a predefined two-dimensional (2D) grid (Fig 1A). A few cases have extended the physical space to three-dimensional (3D) domains to represent morphological features more realistically [29], whereas others have resorted to growing domains that can account for expanding cell populations [30–33]. A notable variation of the generic model is the inclusion of additional species and also of immobile environmental factors. This extension broadens the Turing space of the corresponding two-molecule system while simultaneously removing the requirement of differential diffusivities [34]. The potential of immobile factors for enhancing RD pattern formation had been suggested previously [35, 36] but was particularly highlighted by Macron and colleagues [34], who introduced a sophisticated automated mathematical analysis that directly identified these network topologies along with their respective parameter constraints.

### PI

In contrast to the RD mechanism, there is no universal mathematical representation of PI. The reason is that PI is a phenomenological concept describing the capacity of a cell to interpret its location from its immediate environment [7]. The original theory focused solely on the interpretation of morphogen signals for differentiation by defining threshold values that separate the concentration ranges associated with specified fates (Fig 1B). The premise of PI, that cells can ascertain their location within developing tissue, has been fundamental to the current understanding of morphogenetic patterns and confirmed in experiments, as discussed before. However, no formalism has been established and generally accepted that can be used to verify PI, and PI has therefore mostly been applied as a qualitative descriptor [37, 38]. Some developing systems seem to suggest that the time spent by a cell in a certain position is critical [39]. Specifically, if a signal is restricted to the front end of a growing domain, the duration of exposure to that signal can be inferred as PI. Taken together, PI has not been strictly tethered to morphogen gradients but instead has encompassed a range of mechanisms for designating fate decisions via cell positions.

For instance, there has been a recurring theme of local cell–cell interactions contributing to the acquisition of PI. These interactions could be mediators of PI with varying degrees of influence. At one end of the spectrum, cell–cell interactions were used as noise modulators, providing a spatial averaging effect that enhanced detection of morphogen signaling [40, 41]. At the other end, patterning was derived from a localized, secreted factor affecting cell–cell interactions, without evoking the use of global PI from a morphogen [42]. To what degree cell–cell interactions play an absolutely necessary role in the interpretation of PI is unclear, but the fact that such interactions appear to play a role in various systems suggests that computational approaches are likely to benefit from an implementation at a cellular resolution.

### ABMs

RD models employ relatively simple PDEs, which are sufficient for generic analyses of morphogen gradients and their interactions. However, as soon as the target of morphogenetic modeling is a more realistic, complex space, such as a cell, a blastula, or a developing limb bud, the use of PDEs becomes cumbersome. In fact, it seems almost impossible for analytic PDE models to account for genuinely heterogeneous milieus, in which cells or organisms develop.

An alternative that emerged for such purposes over the past three or four decades is the use of ABMs [43]. The main ingredients of ABMs are autonomous agents that represent some type of entity, such as a molecule, cell, or person. An agent can, in theory, be anything; generically, it is a definable, active or responsive element. The dynamics of an agent may consist of a variety of actions, which must adhere to rules that govern the behavior of the agent. All agents independently make rule-based decisions as they interact with other agents or their simulated environment. Usually these decisions are probability-based, as described in later sections below. ABMs are extremely flexible in their execution and provide an exceptionally powerful framework for the creation of multiscale models (Fig 1C). In particular, they permit the integration of processes across time scales. By their nature, ABMs are particularly adept at capturing spatial phenomena. For example, in an RD system modeled with PDEs, an identical set of equations is solved at each point of an equally spaced grid. Since the reactions are usually cell-mediated, it is implied that each grid point is a cell. This rigidity makes it a difficult task to define a system of equations that account for heterogeneity in a cell distribution, heterogeneous environmental features, or the inclusion of more than one phenotypic state. In contrast, ABMs can easily mimic the patterning of classical RD models but with significantly greater adaptability, such as changes in the environment generated by the agents themselves. Early work demonstrated this option of a crossover between RD systems and agent-based representations in the morphogenesis of ant colonies [44] by implementing local activation and long-range inhibition through memory of the agents. This early work discussed challenges of the “inverse problem” that still exist today for both natural and synthetic systems—namely, how to specify desired emergent Turing patterns by agent rules. Here, the same principles from ant colonies are extended to systems at the resolution of the cell. The properties and history of each cell can be monitored, multiple cell states can be readily implemented, and movement does not have to be constrained to a grid. This flexibility of ABMs permits unlimited options for exploring the dynamics of a complex system. Of importance here is that the ABM framework is very well equipped to simulate morphogenetic events in molecular and cellular detail and to investigate the role of PI in developmental cell fate decisions.

**Fig 2:**
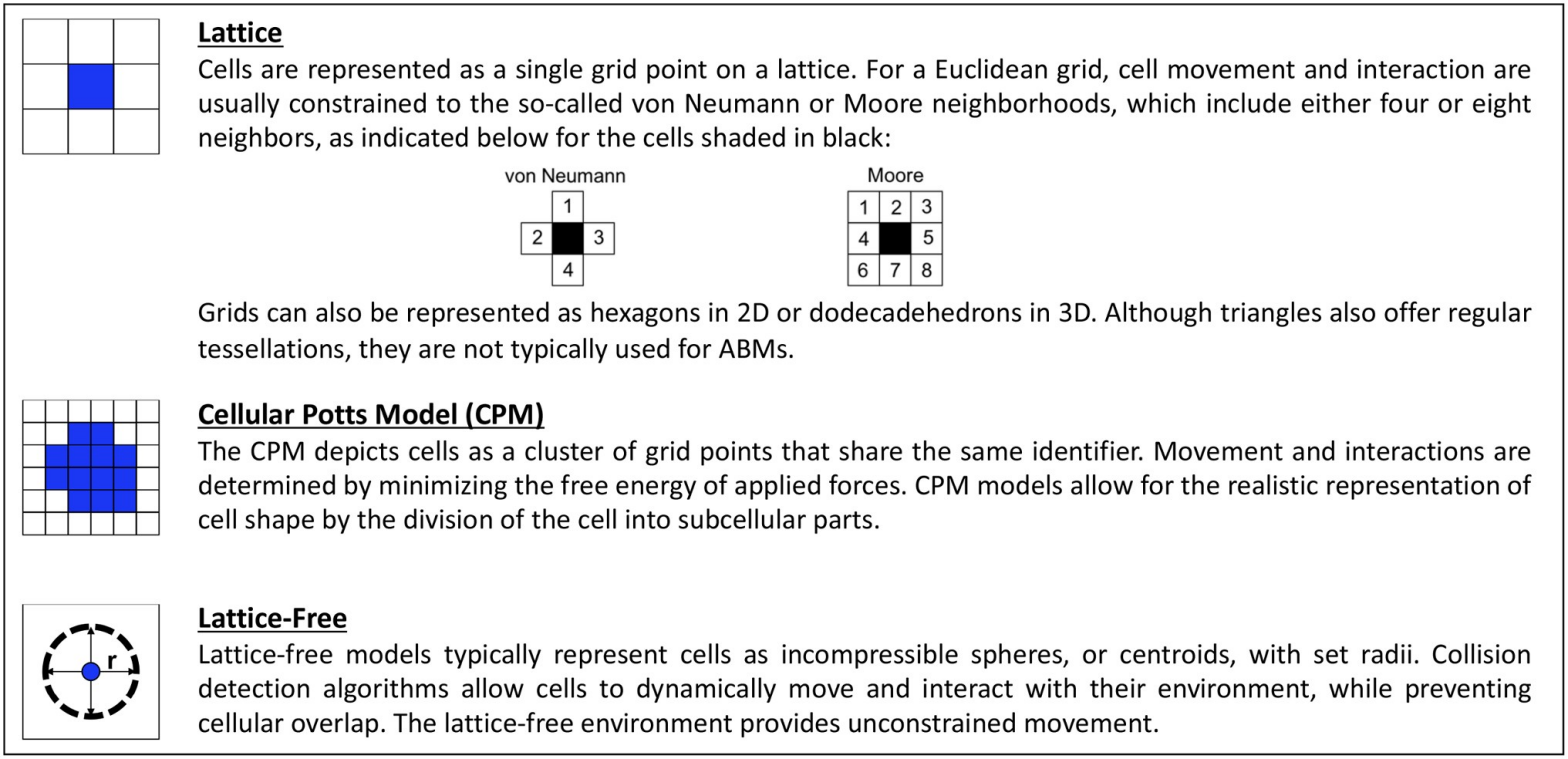
The three most common physical representations of cells in ABMs. Lattice models are generally the least complex, given the constraints to movement and interaction, whereas both CPM and lattice-free models possess varying degrees of complexity depending on the features included in the model. Miniature depictions of the respective images will be placed beside each subsection discussing morphogenetic ABMs in later sections to designate the types of models described. 2D, two-dimensional; 3D, three-dimensional; ABM, agent-based model; CPM, Cellular Potts Model.

The dynamic system represented by an ABM is a collection of constituent parts and rules. For example, in an immunological model, the agents might be various T cells, B cells, and cytokines. In general, the designation of agents is mostly a question of whether the selected parts are capable of replicating the behavior and/or structures of the examined system. This question is directly related to the dominant size scale of the system, which in turn tends to be tied to the timescale of the system. Agents can be portrayed as computational entities in a variety of ways, and the choice of representations is important, as it can affect interactions with other agents and movements throughout the environment. Typical agent representations for cell-based models are illustrated in Fig 2. In the simplest models, agents are simply grid points on a 2D or 3D lattice that have defined properties. The lattice-based representation is the simplest and computationally cheapest. It is also the most limited in terms of mechanistic features and details of cell movement, as movement occurs only to neighboring grid points. The somewhat more complex Cellular Potts Model (CPM) allows agents to consist of often irregularly shaped clusters of grid points that share the same or similar properties. The movement of such a cluster is governed by forces and energy. The CPM is the most flexible for representing irregular cell shapes and movements, and it is also the computationally most intensive model, as it requires forces to be converted to energy. Some of the newer, more flexible models do not rely on lattices anymore. A cell is often represented as a compressible sphere or a centroid, which corresponds to the geometric center of a shape, and a sophisticated algorithm determines movements while avoiding the overlapping of agents. The lattice-free models are well suited for generating 3D organizations and emulating deformations but require more up-front effort for defining realistic details of collision and movement events. As so often is the case in modeling, the “best” representation depends on the questions to be asked, the context of the model, and the availability of data [45].

The rules governing the dynamics of the agents can vary in their degree of abstraction, both across and within models, but within a single model there is usually a focus on either mechanistic or phenomenological descriptions. For instance, a rule may dictate what happens if two agents encounter each other. Such mechanistic rules are established based on empirical data or mathematical representations. An example of the latter might be a random walk model to describe the motion of a molecule by diffusion. In contrast, phenomenological rules are utilized either to mimic an observed behavior or to condense a cluster of mechanistic events into a single representative behavior. For example, cell migration involves a cascade of intracellular signaling, cytoskeleton remodeling, and numerous adhesion forces but is typically modeled as a direct movement response [46]. ABMs that predominantly consist of mechanistic as opposed to phenomenological rules are deemed “bottom-up” as opposed to “top-down” models, respectively. In either case, additional rules can be defined in a manner that the system is able to learn and evolve as a function of past behavior. Thus, agents can become “smarter” in their actions and may exhibit new behaviors as a result of past behaviors and experience.

The agents of an ABM typically move in an artificial environment that represents the biological space of interest in a simplified manner. The simulated environment of an ABM can be as simple or as complex as the research questions demand or the modeler chooses. In the simplest cases, agents move and interact on a 2D grid space within a plane. They may also diffuse through a 3D grid representing a fluid or viscous solution. The environment may be static and passive, in which case the agents interact only with each other, or it can be dynamic, in which case the environment can have an effect on the actions of the agents and/or the agents can change the environment. An example is a predator–prey system, in which the environment could be constant or provide the prey with a feed source that fluctuates dynamically as it grows and is depleted.

Any additional complexity in model design requires extra rules, definitions, and caveats. For instance, in an interactive environment, constant updates are necessary to resolve interactions with agents. Movements and interactions in a 3D system require more attention to the physical properties of the system. Clearly, the computational cost rises significantly for 3D simulations, especially if the entire space needs to be assessed at every iteration of the simulation. As a partial remedy, it is often beneficial to design an ABM in a phased manner: one might begin with a representation of only the most essential features and later add complexity step by step, as soon as the dynamics and the repertoire of possible behaviors of the current model become clear.

A hallmark feature of ABMs is their potential to (re)produce emergent behavior. Expressed differently, the collective decisions of independent agents within a multiagent simulation may result in realistic complexity and behaviors that are not predictable from the governing rules. In effect, the system in such a case exhibits a synergistic response that emerges from the cumulative interactions of its various components. This emergent behavior is not always intuitive or even explainable a priori, especially when the rules are intricate or adaptive. In these cases, simulations are often the only means for identifying and characterizing emergent phenomena within the system [47].

Other benefits of ABMs derive from the realistic nature of autonomous agents and their flexibility, which imposes very few limitations. The autonomy is very important for the definition of rules, because autonomous agents control their own behavior and react to local factors without needing to know the global environment. At the same time, the autonomous agent representation permits heterogeneity, both with respect to agents and rules. The flexibility of ABMs is immense: agent behavior, types of agents, agent interactions, agent adaptation, variability in agent scales, stochasticity, and environmental factors are all unlimited in scope and easily manipulated through corresponding settings of parameter values.

## ABMs for morphogenesis

**Fig 3:**
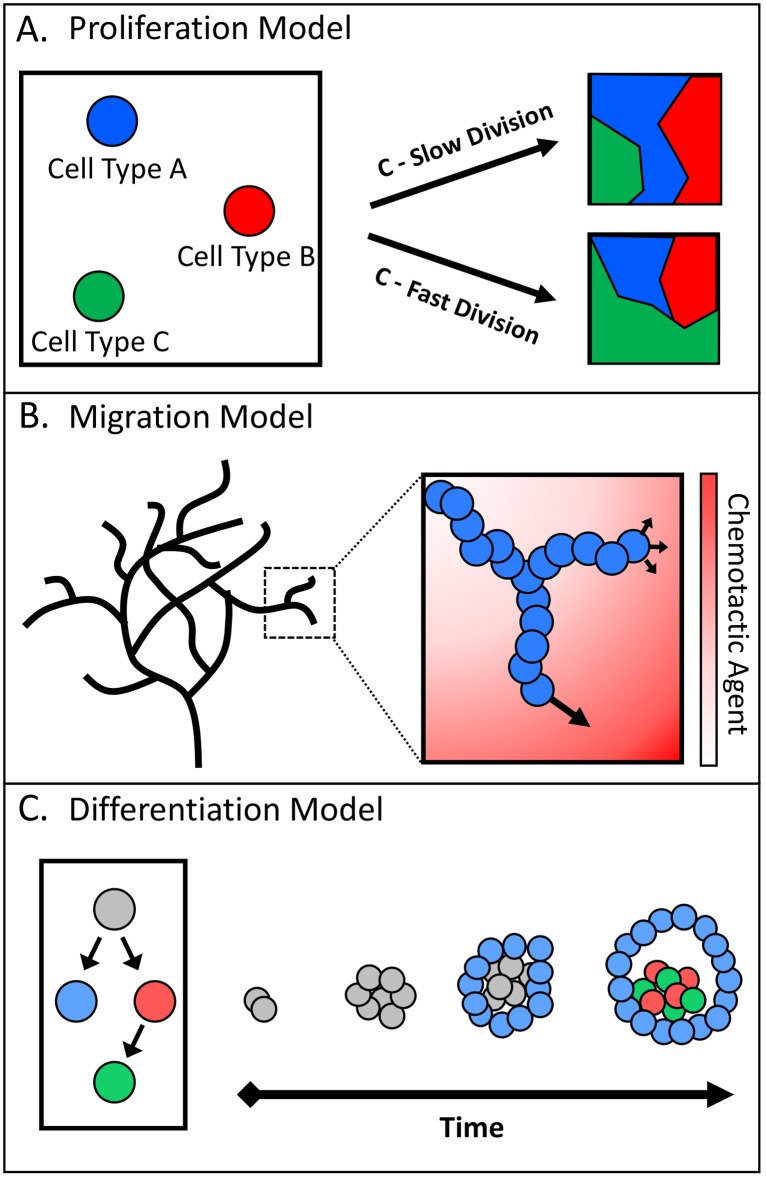
Classification used to categorize three types of agent-based models for morphogenesis; each model class describes the primary mechanism that induces pattern formation. (A) Proliferation models depend on the differential division of cells, either between cell types with variable growth rates, between cell generations, or as an alternative to cell death. The illustration depicts volume exclusion of cell types A and B when cell type C has a fast division rate. (B) Migration models concentrate on directional movement, both in a purely migrational sense and in terms of polarized growth. The patterning of migration models is typically centered on overall morphology, such as the branched network of a vascular network. (C) The focus of differentiation models is on patterning that is reliant on fate change, in most cases following a hierarchy of lineage commitments. The interactions between the various cell types are major determinants for the resultant behavior.

One hallmark of morphogenesis is the coexistence of different types of cells, such as stem cells and differentiated cells. ABMs for morphogenesis use different agents to represent such cells, or various other phenotypes, and study how they organize into the targeted patterns. ABM models of morphogenesis can be broadly organized into three primary categories that denote the predominant mechanism for generating the targeted morphology or type of anticipated pattern in each model: proliferation, migration, and differentiation (Fig 3). As a caveat to this categorization, one must note that there is certainly overlap and that models frequently employ two or all three mechanisms.

Proliferation models place their main focus on differential division rates and on volume exclusion within spatially confined regions to produce patterns (Fig 3a). Furthermore, the patterns are frequently defined by the organization of cell types relative to each other, i.e., the clustering of cell type X surrounded by cell type Y. To generate discernible patterns, these models typically require at least two distinct cell types and/or a method for identifying unique populations. The discussion of proliferation in the next section is divided into subsections based on biological mechanisms that can affect morphological patterning.

Migration models can be classified by the inclusion of directed cellular movement and their emphasis on the gain of cell polarity. A further criterion is the desired morphological pattern of these models in comparison to other patterns. Specifically, the shape and growth of the entire network or system is often more important than the relative location of cell types within the network (Fig 3b). For example, when assessing the formation of the vascular network, one might use the degree of branching and interconnected vessels as a metric, rather than the arrangement of cells within each vessel. The discussion of migration models is therefore split into two subsections representing extracellular factors that can induce migration.

Differentiation models consider systems that are derived from a single cell type but produce patterns or morphologies that contain two or more cell phenotypes. In all cases but one, the system is initially homogeneous with respect to phenotype and gradually gains heterogeneity from differentiation events (Fig 3c). The exception to this strategy is an initialization with heterogeneity, which subsequently requires differentiation into multiple lineages to maintain the desired morphology. Therefore, patterns from this class are described by the relative organization of cell types, which all originate from a stem cell. The subsections highlight two differentiation events that are integral for development: the loss of pluripotency and the gain of heterogeneity in tissue.

If an ABM is designed in a phased manner, the model complexity increases with each sequential phase. As a case in point, the classification into proliferation, migration, and differentiation models itself follows a sequential increase in complexity because of the way cell–cell interactions are involved in these events and how they are encoded. Proliferation models place their main attention on individual cell behavior (division, apoptosis, etc.). Therefore, the most complicated rules that are to be defined only affect the agent performing the rule. Such rules governing individual agent behavior are the easiest to implement because they are self-contained and straightforward to interpret. Migration models account for two additional aspects—namely, the sensing of environmental factors and appropriate responses to such factors. As a consequence, rules are required that describe how an environmental signal is perceived and converted into movement and how this movement is coordinated with neighboring cells. Cell–cell and cell–environment interactions both involve repeated activity updates and, for migration, a balance between adhesion and motility. Differentiation models typically combine the features of proliferation and migration models and additionally introduce differentiation mechanisms, which are usually regulated by environmental factors and/or more complex cell–cell interactions. Thus, the level of detail increases from one section to the next.

### ABMs with proliferation as the major morphogenetic factor

The key feature of ABMs in this category is their capability to specify unique growth rates for each cell type within a simulation. In a developmental context, this variability in proliferation can occur through numerous mechanisms, but the most archetypal instances involve inherent differences in division times between cell types. As a case in point, consider the transition from a pluripotent stem cell to a multipotent progenitor: the genetic distance between the two cell types is relatively short, but the difference between their cell cycle lengths is significant [48]. This fluidity in growth rate as a function of phenotype creates a vast potential for unique spatial organizations that form dynamically over time, even if one only accounts for cell division and the original spatial orientation of each cell type. ABMs provide an optimal mechanism for investigating the effects of proliferation and cellular densities or arrangements during morphological processes.

#### Generational patterning

The first class of proliferation ABMs makes heavy use of cellular invasion waves, which arguably provide the best example for illustrating the capacity of proliferation-based patterning. The phrase “cellular invasion” undoubtedly brings to mind tumorigenesis, but cellular invasion is also an integral part of normal, physiological processes, such as wound healing and morphogenesis [49]. Invasion waves contain two actions, namely cell migration (the invasion), which is followed by proliferation of those cells (the wave). Of particular importance here is the “wave” that occurs after the “invasion.”

As a specific example, the development of the enteric nervous system (ENS) involves the migration of enteric neural crest (ENC) cells to the foregut, where they proceed as an “invading wave” that colonizes the entire gastrointestinal tract [50–52]. Initially, it seems intuitive that the invading wave of cells will maintain an equal composition of progeny from the original population as it grows. However, clonal dominance during ENS colonization has been observed [53, 54]. Experimentally, a single green fluorescent protein (GFP)-labeled ENC cell was added to a population of approximately 8,000 unlabeled ENC cells and fused with gut tissue before the start of colonization [54]. After colonization, large variability was observed in the number of GFP-positive cells contributing to the ENS across experiments, and in one case one-third of the entire ENS was composed of GFP-positive cells. To characterize this appearance of clonal dominance in a population of phenotypically identical cells, a cell-invasion ABM was devised that was capable of tracking cell lineage and generation [54]. The agents were placed on a 2D grid with equal growth rates and with equal ability to move stochastically in the form of a random walk, but only if grid space was available. It is easy to imagine that a cell with a faster division time than others within the initial population would have a competitive advantage and ultimately dominate. However, in this case all cells were assigned exactly the same division time. Tracking cell generations within this model revealed that 50% of the final population could be attributed to the progeny of only a few cells (“superstars”) from the initial population. In other words, despite the identical division time for each agent, a few cells were able to monopolize the available space and create a spatially distinct distribution of their progeny. Analysis of the model mechanics yielded the following: (1) location within the initial population affects the probability of a cell becoming a superstar, but it is not the sole contributing factor; and (2) stochastic competition via volume exclusion is a sufficient mechanism for instigating clonal dominance. Specifically, an accumulation of stochastic movement and daughter cell placement events allows the progeny of a single cell to preclude the growth of other cells by preventing access to grid space. This competitive advantage is reflected in many proliferation models that demonstrate domination through volume exclusion [55–60].

#### Models of balanced growth and death: Apoptosis and homeostasis

Apoptotic events are integral and necessary for shaping tissue during development; two excellent examples are the formation of limb buds and the blastocyst [61, 62]. Combined with proliferation, the balance between cell-specific apoptosis and proliferation allows organisms to maintain morphological homeostasis while at the same time enabling the formation of heterogeneously organized structures.

A popular model system that is highly dependent on apoptotic signals is the formation of small cavities, called acini, in the mammary gland during epithelial morphogenesis of this gland. Several ABMs have investigated acini formation [63–65], but the most recent model stands out because it was implemented on a 3D grid [66]. The 2D model representations of actual 3D systems are informative, but a higher level of abstraction is required for implementing the projection, which often makes model results more difficult to interpret. A good example for this situation is the comparison of proliferation rates: one can easily show that a 2D model requires a slower rate of proliferation than a 3D model to create the same cross-sectional structure. As a thought experiment demonstrating this difference, imagine either a circle or a sphere full of cells. In the former, the maximum number of cells is proportional to circle-radius^2^/cell-radius^2^, whereas for the latter it is sphere-radius^3^/cell-radius^3^. As a consequence of the different powers, the same cell doubling time allows the circle to grow faster than the sphere.

The recent 3D acini ABM [66] begins with a single cell type that expands into the external basement membrane with a designated proliferation potential. As the acinus grows, cells that are not adjacent to the basement membrane receive a signal that triggers apoptosis with a set probability. As cells die over the course of a simulation, the space they occupied becomes part of the acinus lumen. Of particular note from a modeling point of view is that a minimalistic rule set is sufficient to capture the dynamics of normal and aberrant acini morphologies. This rule set slightly modulates the balance between proliferation and apoptosis. It is also important to mention that this delicate growth–death balance only leads to the correct production and maintenance of patterning if two requirements are met: (1) the signals must be relatively equal in magnitude at the population level, and (2) at least one of the signals must be effective in a spatially distinct manner.

The colon crypt is another system that establishes a dynamic steady state between cellular growth and death. The bottom of the crypt is inhabited by a small number of adult stem cells, identifiable by the *Lgr5* marker gene [67], that give rise to a large population of proliferative cells. These proliferative cells both self-renew and produce terminally differentiated cells that make up the majority of the crypt. The patterning capability, which is based on high turnover rates of cells within the intestine, is probably one reason that intestinal and colon crypts have been a popular target system for ABMs. Furthermore, monoclonal conversion, which is synonymous with clonal dominance, is commonly found in crypt systems [68].

**Fig 4:**
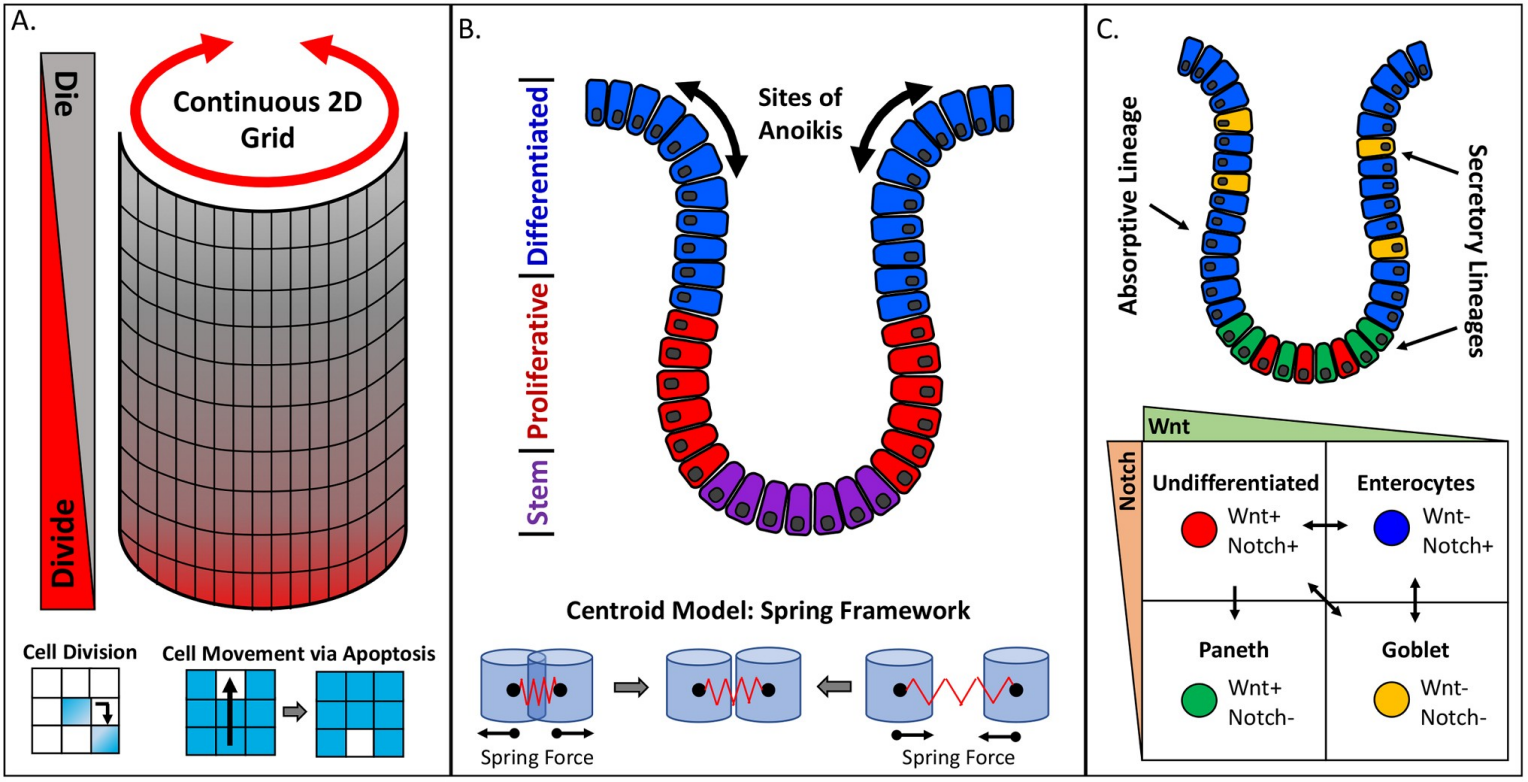
Comparison of three crypt model implementations and agent descriptions. (A) Cells are defined on a continuous 2D lattice such that a cell moving off the right edge of the grid reappears on the left edge. Divide and Die gradients are used to describe the behavior of different cell states in the crypt while movement up the crypt is the result of apoptosis. (B) A centroid model may be employed to investigate the role of crypt geometry on the location of anoikis within a crypt. The proliferative state of a cell is defined by prespecified regions along the crypt, and cell death is solely dependent on the occurrence of anoikis. (C) Centroid model that includes differentiation and dedifferentiation between the four main phenotypes present in an intestinal crypt. Wnt signaling is defined by the position within the crypt, whereas Notch signaling is determined by the phenotype of the cell and its neighbors. 2D, two-dimensional.

Several of the following sections cover a variety of crypt models that illustrate how the same phenomenon may be approached with different types of representations governing the mechanics within the system. Fig 4 contrasts the representations of some of these models. The first crypt ABM was designed on a simple 2D lattice, with cell growth and death defined probabilistically as functions of two preset gradients [69]. The two gradients in this model (termed Divide and Die) are not associated with specific molecules but instead used to provide PI. The probability of a cell transitioning from a quiescent cell to a proliferating cell and then to a terminal cell increases as a cell moves down the Divide gradient. The Die gradient runs in the opposite direction, with the highest probability of cell death occurring at the top of the crypt. Cell movement up the column is defined as a function of cell death, with cells moving upwards to fill any unoccupied space. Similar to the mammary gland acini model, discussed before, the growth and death signals are balanced to maintain crypt size but are also spatially distinct to preserve the cellular organization.

#### Models accounting for external proliferation cues

Numerous diffusible factors in the extracellular environment can influence the proliferation of a cell. These factors range from something as simple as the availability of nutrients to growth-specific proteins that are aptly termed growth factors. Indeed, many growth factors have been classified as morphogens because of their ability to promote proliferation and modulate differentiation potential during development [70, 71]. Many examples of ABMs rely on such morphogens.

The development of the genital tubercle (GT) provides a clear example for the critical role of morphogens. GT development is identical in males and females until stage embryonic day (E)15.5 of embryogenesis, when the male GT is exposed to androgens. The androgen signal promotes proliferation of both mesenchymal and endodermal cell types in the GT, resulting in sexual dimorphism. An ABM of GT morphogenesis was implemented in the software CompuCell3D using a CPM that captured adequate movement and interaction dynamics [14, 72]. A simple signaling network was integrated into the model to account for the three principal morphogens that direct GT development: SHH, fibroblast growth factor 10 (FGF10), and androgens. SHH and FGF10 induce growth in mesenchymal and endodermal cells, respectively, whereas androgens enhance sensitivity of urethral plate endoderm and preputial mesenchyme to FGF10. In the model, SHH was secreted by endodermal cells at a constant rate, thus stimulating the secretion of FGF10 in mesenchymal cells in a concentration-dependent manner. Androgens were assumed to permeate the system at a set time point (E15.5), causing an instantaneous change to the FGF10 sensitivity in all affected cells. The model was initialized with an idealized geometry of the tubercle at E13.5, containing five distinct cell types. Simulations using this model were able to recapitulate the sexual dimorphism that occurs during GT development by modulating proliferation in response to androgen signaling.

During puberty, the mammary gland undergoes extensive proliferation and ductal morphogenesis [73]. Previous studies had shown that exposure to ionizing radiation before or during puberty significantly increases the risk of developing breast cancer in women by causing an increase in the mammary stem cell population [74]. To test the mechanisms eliciting the morphogenetic changes observed after puberty, an in vitro and an in silico ABM were defined [56]. The in vitro model identified transforming growth factor β (TGFβ) as a major activator of growth during ductal morphogenesis, whereas the in silico model addressed the mechanism by which radiation could modulate the TGFβ-induced growth to produce a larger stem cell population. The ABM was contained on a 2D grid and included three cells types: bipotent progenitor cells, terminal basal cells, and terminal luminal cells. The progenitors had the option to divide symmetrically or asymmetrically, producing two stem cells or one stem cell and one basal or laminal cell, respectively. Furthermore, a dedifferentiation mechanism allowed terminal cells to convert back to bipotent progenitors. The model parameters associated with the probabilities that these events occurred were fit using experimentally determined distributions of the cell types as the objective metric. These experimental distributions were obtained from the in vitro model using TGFβ to stimulate growth, with and without irradiation. By comparing the calculated best-fit parameters between these conditions, the likely mechanism precipitating the increased stem cell population was identified as enhanced self-renewal in response to TGFβ.

Although differentiation was an important component of this model, it played a less vital role than the proliferation rates for yielding the desired patterning. Specifically, the majority of daughter cells actually originated from a parent of the same phenotype rather than via differentiation of a progenitor cell. This situation arose because differentiation in the model was coupled to cell division, with the consequence that the rate of differentiation could never surpass the rate of cell growth. Furthermore, because growth could only occur where space was available, the growth of progenitor cells was quickly hindered by other cell types through competition due to volume exclusion.

#### Models of growth directed through mechanical forces

Mechanical forces are involved in nearly every aspect of morphogenesis, albeit with varying degrees of influence. At the macroscopic scale, mechanical forces can shape and organize tissue in response to the cumulative effect of numerous forces at the microscopic scale [75]. In particular, cell adhesion molecules allow cells to bind together and to their environment, and these adhesion forces affect how the population expands outwards as cells divide.

Up to this point, the ABMs discussed have been constrained to lattices, and in many cases, division was only allowed to occur when space was available. Although lattice structures are useful and computationally efficient, they offer limited spatial resolution and lack the ability to describe intercellular forces. Furthermore, the division constraints are contextual and require scrutiny: a constraint could represent contact inhibition or quiescence, but it also frequently creates an artificial scenario in which mitosis is restricted to the edge of a cell cluster. Centroid models overcome these limitations by allowing lattice-free movement. A centroid model represents each cell as a central point that is connected to neighboring cells by stiff springs. If a cell comes within the confines of another cell, whether by movement or division, a collision occurs. The collision produces a force that moves both cells to prevent overlap and, if necessary, propagates this movement through the population.

An effective demonstration of a centroid model is again a colon crypt model [76]. This particular ABM sought to probe the mechanisms behind crypt anoikis, i.e., programmed cell death in response to detachment from the basement membrane. In this ABM, cellular agents grow and move freely along a 3D rendering of the crypt membrane, experiencing cell–cell collisions and an attachment force to the membrane. In the biological system, anoikis typically occurs at the top of the crypt, and the authors hypothesized that the crypt geometry and attachment forces were the crucial factors for simulating the process. By modulating the attachment forces, the localization of anoikis events could be replicated, and the system was able to self-regulate the rate of anoikis, thereby maintaining homeostasis. Results like these highlight the advantage of lattice-free models for investigating behavior that emerges as a function of interaction forces, especially within complex geometries.

When pondering mechanical forces during proliferation, one might also consider the effects of external resistance. It is known that the extracellular matrix (ECM) can act as a scaffold for growth; a pertinent example is the basement membrane of the second crypt model. The ECM can also act as a boundary or an anchoring point [77]. The stiffness and elasticity of ECM are highly variable, and these properties influence the response of cells and their organization during growth.

An example is adipose tissue, which consists of adipocytes clustered into distinct lobules by ECM, with variable morphologies. Using a lattice-free ABM platform, the self-organization of adipose tissue was investigated with adipose cells modeled as growing spheres and ECM fibers as short lines that could cross-link together [55]. Each ECM fiber had a defined, constant unit strength, with larger strands of cross-linked fibers able to exert more force on neighboring adipose cells through the fiber network. Additionally, the adipose cells were able to exert pressure on the ECM network to prevent compression as the available space for growth decreased. To explore the dynamics of lobule formation, the linking–unlinking frequency (*ν*_*d*_) of the fibers was investigated, which revealed that adjustments of this quantity could result in three distinctive morphologies. Since modulation of *ν*_*d*_ was representative of the degree of ECM restructuring, the three morphologies were regarded as consecutive “phases” that developed over time.

### ABMs with migration as the major morphogenetic factor

Migration events are commonplace throughout development and pivotal for the morphogenesis of numerous tissues [78]. The forces behind cell motility are predominantly mechanical, but the signals that trigger and direct migration can be mechanical, chemical, electrical, or all three. As mentioned previously, cells in vivo are experiencing a multitude of forces from the environment and neighboring cells. Furthermore, expansion of a cell population can lead to passive movement of the cells in response to physical interaction effects (collisions, boundaries, etc.). In contrast to passive movement, migration involves the active generation of forces by a cell to induce movement, usually in response to an external stimulus. Concurrently, cell polarity accords directionality by establishing a leading or front edge during movement. The ABMs discussed in this section emphasize two key mechanisms whereby heterogeneous environmental factors can direct and coordinate cell movement.

#### ECM influence on migration

Cell migration during development exhibits a collective form of organization whereby cell populations are able to traverse long distances as coordinated groups. The emergence of this behavior requires one or more environmental cues that guide the migration of each cell. One source of directional information for migrating cells is the variable composition of adhesion and signaling proteins within the ECM [79]. The organization of ECM components is dynamically modulated by local cells that can secrete and degrade ECM macromolecules [80]. The resultant remodeling of the ECM can bias migration [81] and even reinforce specific migration paths [82].

The gastrulation in amphibians provides a representative example of collective migration that is dependent on an ECM component: fibronectin (Fn) is essential for mesendoderm cells to migrate as a sheetlike cluster across the inner surface of the blastocoel [83, 84]. As the cells progress, they bind to the ECM and exert traction forces via integrin–Fn interactions, which restructure the ECM in the wake of the first migratory cells [85, 86]. In addition, mesendoderm cells exhibit “shingling” behavior: cells overlap each other and maintain cell–cell adhesion, mediated by cadherins, during movement.

An ABM, reminiscent of a standard CPM, was developed to investigate the capability of these interactions to guide coordinated migration [87]. Specifically, each cell is represented by a 3 × 3 grid on a 2D lattice of Fn, in which the center represents the cell body and its Moore neighborhood the cell’s “edges.” The edges of adjacent cells are allowed to overlap and produce an adherence force that mimics shingling behavior. In the model, the Fn matrix is initialized with randomly distributed concentrations along the x-axis but with a mild gradient along the y-axis. The restructuring of the matrix is modeled by a decrease in Fn concentration each time a cell moves through it. To determine the magnitude and direction of movement, the Fn gradient is calculated along each edge, with larger forces being generated by larger Fn differentials. A similar force is calculated for each pixel overlapped by another cell, and the net force vector is computed as the sum of both forces. This customized framework is able to achieve cellular movement along the Fn gradient but cannot capture the observed, coordinated sheetlike clustering. In particular, the balance between integrin and cadherin turns out to be disproportionate along the edge of the cell colony. To remedy the situation, the authors introduced an intracellular feedback network based on Wnt/B-catenin signaling, whereby integrin binding triggers cadherin production, which in turn equalizes the integrin and cadherin forces and allows a sheetlike migration to occur.

A second representative example in this category is the development of the neocortex, which involves an interesting pattern of migration from the intermediate zone (IZ) toward the marginal zone (MZ) [88–90]. The neocortex consists of six distinct layers of pyramidal neurons. The migrating cells form a growing cortical plate (CP) between the IZ and MZ, one layer at a time. Cortical layer VI is the first layer of cells to form and is positioned closest to the migration source (IZ), with each sequential layer migrating over previous layers in an inside-out manner and increasing cortical thickness [82]. Reelin is an ECM glycoprotein that is essential for the proper formation of the neocortex [91–93]. In fact, the cortical layers in a Reelin-null mutation mouse (“reeler” mouse) are inversed, with layer VI becoming the most superficial layer [94]. However, the exact function of Reelin and its effect on migration through the CP have been debated [88]. To explore the different hypotheses regarding the role of Reelin during cortical development, a set of migration ABMs was generated with various rule sets to reflect each proposed mechanism [95]. It is unnecessary to describe each individual rule combination, but in general, the following held for these models: each layer of cells was introduced to the system independently and uniquely colored, one row at time; furthermore, cell movement and the conversion to an immotile state were defined as functions of Reelin. Extensive model testing revealed that it is possible to isolate a rule set that very closely mimics the biological system (Fig 3a). This inference of rules was accomplished by comparing the model output (i.e., the colored cell distributions of CP) against the known behavior in reeler mutants and in a Reelin-dependent mutant, *Dab1* [96]. Other ABMs involving ECM-mediated migration include [97–99].

#### Models accounting for chemotactic cues

A widespread mechanism driving morphogenesis is a branching process. Indeed, this process can be found across multiple organ systems, including the lungs, kidneys, and vasculature [100–103]. In each of these tissues, the branched morphology is achieved through the recurrence of three events affecting the bud or vessel: formation, extension, and splitting. The branching morphogenesis in each of these organ systems is sensitive to the spatial distribution of a key morphogen, namely, FGF10, glial cell–derived neurotrophic factor (GDNF), and vascular endothelial growth factor (VEGF) for lungs, kidneys, and vasculature, respectively. Multiple mechanisms have been proposed for initiating the spatial distribution of each morphogen, including ligand-receptor-based Turing mechanisms for the lung and kidney systems [19, 104]. In these cases, the morphogen induces expression of its receptor, which correspondingly increases sensitivity to the morphogen. This receptor–ligand cooperativity creates regions of high receptor density with enhanced morphogen activity that are interpreted as locations for branching events. A more common approach for modeling branching events is the consideration of a morphogen as both an inducer of proliferation and as a chemoattractant. High concentrations of the chemoattractant polarize the tip of a growing bud or vessel and cause proliferation in the respective direction. As the network expands, the vessel/bud cells consume the morphogen and establish a gradient that directs future growth and branching events.

A CPM of a ureteric bud in the kidney was compiled to determine the relative influence of mechanochemical factors on the observed branching morphology [105]. Specifically, this model assesses the factors affecting a single splitting event within the kidney. Model analysis revealed that the morphology is most strongly influenced by two model parameters representing the strength of chemotaxis and the proliferation rate. In fact, if chemotaxis and proliferation are perfectly balanced, the physiological morphology is achieved, whereas skewing the ratio leads to pathological morphologies.

A different role of branching morphogenesis can be found at the network level. A good example is a recently published 3D model of vasculogenesis that delves deep into the mechanical aspects of these branching processes [106]. In contrast to what one might expect, the morphogen (VEGF) is not implemented in the model as a direct activator of proliferation. Rather, VEGF acts exclusively as a chemoattractant that stimulates migration of vessel tip cells. Nonetheless, proliferation is important in this ABM. It is stimulated through mechanical stretch forces generated by the “pull” force of chemotactically migrating cells, which has indeed been experimentally observed in vascular endothelial cells. The mechanism by which the stretch affects proliferation in these experiments consisted of up-regulating the VEGF receptor. The ABM for this system uses a lattice-free, centroid description of two cell types: tip cells and vessel cells. Both cells types are subject to multiple local forces that govern their behavior (Fig 3b). Tip cells experience a chemotactic force along a VEGF gradient, a persistent force that results in the tendency of cells to continue moving in the same direction, an environmental drag force, and interaction forces from neighboring cells. The vessel cells are exposed to interaction forces, environmental drag forces, and an angular persistence force that stabilizes and corrects possible buckling caused by cell division. Furthermore, mechanical stretch and compression of vessel cells are used to regulate proliferation and sprouting events, respectively. The complex rule set of this ABM is unique compared to other models of branching morphogenesis, which typically assume chemical dominance in extension and splitting events. The topics of angiogenesis and vasculogenesis have been extensively researched and reviewed previously [107], with copious ABMs for both phenomena [103, 108–117].

### ABMs with differentiation as a necessary morphogenetic factor

Morphogenesis and differentiation are highly interdependent. All organs and tissues are composed of heterogeneous assemblies of cells, and the acquisition of phenotypic heterogeneity through differentiation often occurs concurrently with the gain of organization. Thus, although differentiation is not as directly involved in shaping tissue as proliferation and migration are, it is clearly essential for morphogenetic events. The following describes representative ABMs that focus on (1) initial differentiation from the pluripotent state and (2) differentiation within tissues.

#### Gain of organization during loss of pluripotency

The loss of pluripotency at the beginning of development prefaces nearly all instances of morphogenesis. As development proceeds, pluripotent cells differentiate into the three germ layers and eventually every somatic cell type. These initial fate decisions are crucial for embryogenesis and require coordination between the processes that maintain pluripotency or designate cell fate [118]. The transcription factors governing the pluripotent state can be reduced to a “core” network consisting of octamer-binding transcription factor 4 (Oct4), sex-determining region Y–box 2 (Sox2), and Nanog [119–122]. These key transcription factors dynamically regulate their own expression and have essential roles in directing self-renewal and fate specification [123–126]. The self-renewal aspect is of particular importance for the study of pluripotent cells in vitro. Pluripotent cells are only transiently present in vivo, and they quickly acquire germ layer fates. Therefore, sustaining expression of the key transcription factors associated with self-renewal is necessary for the culturing of pluripotent cells in vitro. Typically, this task is accomplished by the addition of exogenous factors such as leukemia inhibitory factor (LIF), which indirectly activates Sox2 and Nanog expression in mouse embryonic stem cells (ESCs) [127, 128]. Since the discovery of means for maintaining pluripotent cells in vitro [129–132], the opposite has become very alluring as well: harnessing the specific organ- and tissue-forming potential of these cells.

Aggregates of pluripotent ESCs can serve as powerful in vitro platforms for studying morphogenesis and early differentiation events, both experimentally and with ABMs. In a landmark study, White and colleagues found that spontaneous differentiation of these aggregates produces transitional patterns as pluripotency is gradually lost [133, 134]. Specifically, spontaneous differentiation was instigated by culturing the cell aggregates in the absence of LIF. The transition out of the pluripotent state was monitored by collecting representative images of the pluripotency-regulating transcription factor Oct4 expression within the aggregates over the course of differentiation.

To investigate the emergence of patterns, a 3D ABM utilizing the centroid schema was constructed. The ABM simulations considered two cell types (Oct4+ and Oct4−), with the initial population consisting entirely of Oct4+ cells. Three mechanisms for triggering state change were explored: stochastic processes, juxtacrine signaling, and paracrine signaling. In total, seven unique rule sets for governing differentiation were formulated and compared. In general, for both juxtacrine and paracrine signaling the Oct4+ cells were modeled as the source of inhibitory factors, whereas Oct4− cells acted as the source of differentiation activators. To compare the experimental and simulation results, network metrics were extracted from a training set of computationally generated pattern classes. Principal component analysis (PCA) was applied to the multivariate set of metrics calculated from the training set networks and mapped onto a dimensionally reduced latent space. By applying the same PCA transform to metrics calculated from the experimental and simulation data, spatial patterns of Oct4 expression could be evaluated as a function of proximity to each pattern class within latent space. This strategy facilitated a direct and quantitative comparison between the simulation results and the experimental data, thus identifying the state-change mechanism best able to describe the observed patterning.

**Fig 5:**
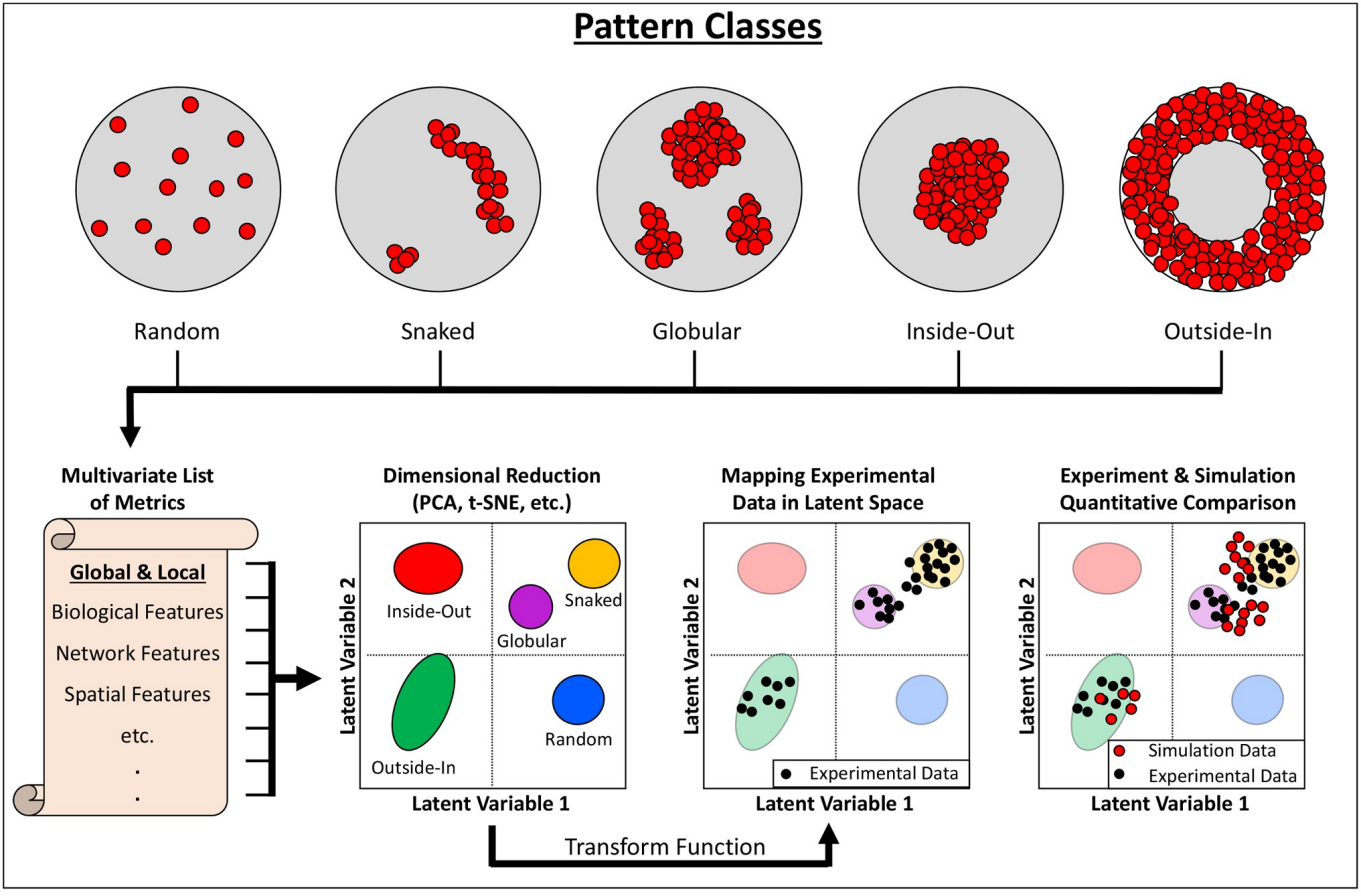
Overview of the analysis of spatial features using a combination of network analysis and dimensional reduction techniques. A set of metrics is calculated or extracted from a series of pattern classes that depict typical cell organizations within the system—here, using the defined pattern classes from [133, 134]. The selected metrics should be equally represented in the simulated and experimental systems. Dimensional reduction techniques allow the multivariate data to be condensed to a few axes, ideally separating the defined pattern classes into distinct regions of the latent space. A transform function trained on the pattern class data can then be applied to metrics calculated from experimental and modeling results, mapping both into latent space and compared to the locations of the pattern classes. PCA, principal component analysis; t-SNE, t-distributed stochastic neighbor embedding.

As with other studies, this model investigation faced a generic challenge of agent-based modeling, namely the unbiased quantification of results. Here, model validation was approached by assessing population-based and phenotype-specific metrics that were able to quantify spatial features rather than relying on visual comparison. In fact, the specific methodology of this study may serve as a generic means of validating ABMs by comparison of spatial characteristics, which is particularly important in morphogenetic studies. For example, the same network-based analysis was used to examine the evolution of spatial patterning during cichlid gastrulation in vivo [134]. Fig 5 provides an overview of this approach for comparing spatial patterning between simulation and experimental systems.

ABMs have been used to elucidate another aspect of early differentiation in vivo, namely the loss of pluripotency and the concomitant morphogenesis of a blastocyst [135]. This process of blastocyst formation is remarkably robust and entirely self-contained, receiving no external maternal information. Rather than attempt to delineate every signal and interaction, the authors of this ABM decided to define overarching rules in a top-down approach. The question was simple: What is a minimalist set of rules that can adequately capture the complexity of blastocyst formation? Extensive exploration of various ABM implementations suggested that four rules, derived from four main regulatory events, were sufficient to recreate the structuring of the blastocyst. The first rule targets polarity at E3.0, thereby establishing the inner cell mass (ICM) with trophectoderm cells along the periphery. The second rule mimics FGF signaling at E3.5 and creates a salt-and-pepper distribution of epiblast (Epi) and primitive endoderm (PrE) cells. The third rule causes lineage segregation of the Epi and PrE cells through differential adhesion. Finally, the fourth rule causes apoptosis of any PrE cells that were not segregated properly from the Epi cells. The model was defined as lattice-free and implemented in 2D, in which the success rate for achieving correct blastocyst formation using these four rules was 79%. In a slight modification, the model was also validated in 3D with minor changes to account for differences in the number of nearest neighbors.

Several biological insights related to the FGF/extracellular signal–regulated kinase (ERK) pathway emerged from this work. First, the specification of Epi and PrE cells in a salt-and-pepper pattern resembles a Turing-like mechanism. Second, FGF4 is secreted by ICM and Epi cells and inhibits Nanog. Third, Nanog and Gata6 mutually inhibit each other, which effectively creates a positive feedback loop outside the steady state. The consequence is a local intracellular amplification mechanism which, in conjunction with a global inhibitor, produces a spot pattern. Furthermore, the initiation of FGF/ERK signaling appears to be invariant to the number of cells within the embryo and is instead dependent on the time since fertilization. As experimental validation, scaling experiments were conducted whereby mouse embryos at the 8-cell stage, before trophectoderm specification, were merged into 16-cell and 24-cell embryos. Blastocyst formation proceeded normally in these double- and triple-embryos, albeit with approximately 2- and 3-fold more final cells, respectively. Of important note, the ratio of PrE to Epi cells was maintained within the ICM despite the scaling, indicating that the patterning mechanism does not rely on—nor is sensitive to—the quantity of cells within the ICM. Experimental evidence of the time dependence was provided by the response to transient inhibition of ERK: the PrE/Epi ratio decreased in proportion to the duration of inhibition. The model predicts that the time dependence of these initial fate decisions is due to a necessary accumulation of FGF or a similar signaling component from the onset of fertilization to the point of activation.

#### Multiphenotypic tissue models

As a representative ABM in this category, we return again to crypts but focus here on intestinal crypts, which share the same physiological structure with the colon crypt but contain an additional cell type, called Paneth cells [136]. In the previous colon crypt models, cell types were not explicitly defined beyond their proliferation capacity. Here, the individual cell types are considered along with their type-specific interactions; they include undifferentiated (stem-like) cells, secretory progenitors, secretory goblet cells, secretory Paneth cells, and enterocyte progenitors. The model has some similarities with the second crypt model discussed before in that it is a centroid model in which each cell experiences adherence forces to the basement membrane. In addition, the model here includes active migration, extracellular signaling, intercellular signaling, and differentiation. Migration is implemented as a constant upward movement for all cell types, except for Paneth cells that migrate downwards. The extracellular signaling molecule Wnt is considered to be a function of local crypt curvature. As a consequence, Wnt exhibits a constant gradient, with the highest concentrations at the bottom of the crypt. Intercellular signaling is defined to be a function of Notch, where undifferentiated and enterocyte progenitor cells produce the receptor, and secretory lineage cells produce the ligand. In addition, Notch signaling acts as an inhibitory signal for secretory cell differentiation in neighboring cells. Therefore, the secretory cells inhibit the differentiation of nearby cells into secretory cells in a process termed lateral inhibition [4]. The various lineage progressions between cell types, including dedifferentiation, are summarized in Fig 4c. The complex model is capable of replicating numerous biological phenomena reported in the literature, such as recuperation of the undifferentiated cell population after ablation, and predicts that a similar recovery is possible for each functional cell type within the crypt. This prediction reflects the primary focus of the model to emphasize the role of cell–environment interactions in establishing the functional phenotype of a cell. Specifically, it stresses that fate decisions are fluid and progenitors are capable of interconverting or dedifferentiating if exposed to the right set of cues.

Many morphogenetic events include populations with diverse sets of transitioning phenotypes and cell type–specific interactions. As the number of interactions expands, it becomes more difficult to intuit the role of any single interaction within the system. The capability of ABMs to capture the emergence of system-level features in response to the addition or removal of single interactions is a potent tool for probing developmental events. Indeed, other differentiation-focused ABMs have explored a myriad of developmental processes, such as somite formation [137], establishment of the germline in *Caenorhabditis elegans* [138, 139], and others [140–145].

### Manipulation of morphogenesis based on ABMs

Morphogenesis has been studied for over a century, but a modern goal of morphogenesis research has become the manipulation of developmental mechanisms for purposes of targeted tissue engineering. Specifically, the short-term goal is to achieve functioning organ systems by replicating environmental conditions that regulate the targeted morphogenetic events in vivo. This modern line of research fundamentally asks questions regarding the number and character of conditions that are sufficient to emulate the morphogenesis of any given tissue. ABM provides a unique platform for mimicking realistic single-cell behavior at the tissue level in response to spatially and temporally diverse signals. Indeed, agent-based modelers hope that it is possible to derive the necessary conditions and interactions by iteratively simulating organogenesis from its inception under slightly altered rules and conditions. An excellent illustration of this pursuit is the modeling work of Setty and colleagues on early pancreatic organogenesis [146]. Their ABM consists of three main components: a reactive system engine for running the model, a front-end animation, and a graphical user interface (GUI) for mathematical analysis. The reactive system engine permits user interactions during runtime, such as pausing a simulation or adding or removing stimuli. In parallel, the front end provides a 3D animation of the model during runtime, and the GUI analyzes the system. This implementation permits real-time analysis and manipulation of the simulated system. The obvious advantage of this approach is its ability to gauge the impact of specific conditions as they emerge.

The agents in this ABM consist of three interacting components: the cell itself, a nucleus, and a membrane. The cell component makes growth and fate decisions, using information from the nucleus and membrane. The nucleus contains a set of genes that may be expressed or silent as a function of the environment and the cell state. The membrane responds to environmental factors, such as the binding or releasing of extracellular molecules, which triggers cell movement. The various features of the model are defined in a statechart, which designates the cell types and their independent rules for cell–cell and cell–environment interactions. With these methodological settings, the model is able to recapitulate 2D histological features of the developing pancreas with high fidelity and generates results that are visually similar to 3D histology samples. Although quantifiable validation is limited, the model is capable of creating tissue-scale morphological features that depend solely on single-cell decisions in response to environmental cues. Thus, with appropriate caution, this type of model can be a useful tool for determining and fine-tuning conditions that are necessary for deriving complex tissue structures in vitro.

### Limitations of ABMs for morphogenesis

Like all modeling approaches, ABMs have clear strengths but also germane weaknesses. The description of ABMs in the previous sections has demonstrated that a particular strength of ABMs is the relative ease with which specific hypotheses can be explored and tested. At the same time, some modeling issues that might seem straightforward at first are actually quite difficult to assess for ABMs.

#### Inverse questions

A typical question for an ABM is, Is this hypothesized mechanism sufficient to generate an observed pattern in time and space? Although such a question can often be answered, a seemingly similar question is comparatively very difficult to assess with ABMs: Is this hypothesized mechanism actually driving the biological phenomenon, and/or are there other mechanisms that are operating in parallel? More generally, “forward simulations,” which mimic “what-if” scenarios, are natural for ABMs, whereas it is difficult to address “inverse” questions, which attempt to determine feature representations from high-level data. For instance, it is difficult to infer the mathematical format of a mechanism, such as a growth or migration process. It is similarly difficult to answer questions such as “how many steady states can this system have?” or to characterize such steady states, especially if they are unstable. Along the same lines, it is sometimes difficult to answer questions like “is it possible for this system to exhibit a particular behavior?” Nonetheless, it is not entirely infeasible to characterize ABMs with rigorous mathematical analysis. For instance, a very thorough analysis of CPMs demonstrated some shortcomings with the depiction of cells with subcellular parts [147]. Although a significant portion of the analysis was dependent on the infrastructure of CPMs, the approach itself highlights the potential for applying mathematical and model theory from other fields to answer some of these questions on an individual model basis.

#### Parameter fitting

A second complex of challenges pertains to fitting parameters. In particular, the designation of rules does not always lend itself to parameters that can be directly measured. In some cases, parameter values may be inferred from experimental data, if they are independent of other parameters, but when multiple parameters are interdependent, they have to be fit simultaneously. However, ABMs are not naturally amenable to parameter estimation algorithms, and the typical steepest-descent methods or genetic algorithms face issues with ABMs. Two factors that further complicate the situation are the stochastic nature of ABMs and the phenomenon of emergence, which can lead to high parameter sensitivities—i.e., they can lead to large fluctuations in system behaviors in response to small changes in parameter values. As a consequence, typical parameter estimation techniques would require very large numbers of iterations for each parameter set, which would incur significant computational costs. Addressing these difficulties, new methods for parameter estimation in ABMs have begun to appear [100, 148].

#### Model validation: Comparison to experiments

Specifically with respect to morphogenesis, the usual output from an ABM is a set of agent-objects that contain spatial and state information at a cellular resolution. However, the patterns and morphological structures that are being targeted are formed at the cell population level. It is quite easy to qualify similarities between experimental observations and simulation results by visual comparison, but some form of quantification is necessary for model validation and the ability to make definitive claims. The derivation of such quantifiable metrics, especially those that characterize spatial features, is a nontrivial task. Spatial metrics need to be able to classify the same types of often irregular features and denote them with similar representative values for both experiments and simulations. Because of difficulties associated with this task, the use of spatial metrics for validation has been relatively rare, which is surprising for models targeting morphogenetic events. Instead, it has been more common to attempt model validation through easily quantified population-based metrics. Although population metrics do provide some connection between the simulations and the true system, they are not optimal for models that are designed to explain or predict pattern formation.

Two methods that are used most frequently for evaluating pattern formation are image analysis and network analysis. In the former, the simulated agents are converted into images that match the experimentally obtained images, whereas in the latter, the experimentally obtained images are converted into digital networks that have the same format as the agents. In both scenarios, representative features are extracted from data or calculated. For instance, cell locations are determined by identifying nuclei in the experimental images. If the critical features cannot easily be identified, customized algorithms are needed that allow pattern classification without the need of explicitly defined metrics [149, 150]. The acini model, for example, demonstrates the use of image analysis for model validation, with images acquired at multiple z-planes for 3D comparisons [66]. An excellent example of validation per network analysis can be found in the ESC aggregate model [134]. Finally, the combination of a dimensionality reduction technique, such as PCA, with either of these analysis methods can be an effective way to visualize dynamic changes in patterning in addition to quantification [151].

#### Computational costs

The computational cost of ABMs can quickly become intractable as the number of agents multiplies within a simulation. The most common approaches for overcoming the computational costs associated with ABMs center on the abstraction of agents. For instance, the abstracted agents can represent clusters of cells within the biological system but are depicted as individual agents in the simulation. This form of abstraction is usually static over the course of a simulation, and all agents are scaled equally while maintaining the spatial features of the target system, i.e., [72, 136, 146]. It is also possible to cluster agents dynamically into larger “meta-agents” during a simulation [152]. This adaptable abstraction can decrease the number of iterations at each time point and thus accelerate the computation time but requires periodic checks of each meta-agent.

Other methods for improving the functionality of ABMs in morphogenetic research involve technological improvements, with one example being graphical processing unit (GPU) parallelization [153–155]. An evident advantage of parallelization is the ability to simulate much larger cell populations without compromising computational time. Thus, more realistic tissue-scale models can be produced even on regular desktop computers. An added benefit is that models simulating fewer cells can run faster, thereby potentially eliminating some of the issues regarding parameter estimation. However, parallelization requires certain constraints that can either limit functionality entirely or reduce certain functionality, based on the user’s programming knowledge. At this point, ABMs seem to be gaining the capacity to model tissue-level morphogenetic events, but given the paucity of specific examples so far it is hard to predict how the various interactions will translate into a parallel infrastructure.

## Discussion

Morphogenesis is a paradigm for the benefits of merging traditional, reductionist biology with some of the newer concepts of experimental and computational systems biology. Understanding morphogenesis requires the very detailed elucidation of individual processes, but it also depends critically on solid knowledge of the dynamic interactions among these processes. ABMs are unique in their ability to investigate and integrate combinations of processes and their respective dynamics. We demonstrated this flexibility here with the large diversity in approaches for the various aspects of morphogenesis that have been analyzed with ABMs so far. As a notable example, the three different crypt models addressed the same biological system with three unique and genuinely different models that each answered a specific question. The vasculogenesis model expanded upon the typical chemical models of branching morphogenesis by also considering the numerous mechanical cues that affect the direction and degree of vessel growth. In contrast, the blastocyst model condensed the significant knowledge base of early development down to four simple rules that were still able to accurately describe the system.

ABMs are useful even for the exploration of poorly studied systems that lack sufficient data. They can be developed initially with rather coarse, top-down, behavioral-driven rule sets that ultimately generate hypotheses regarding those processes that are most influential for producing a given morphology or pattern. These hypotheses can in turn guide the design of laboratory experiments that increase the likelihood of identifying key events or pathways within the system. Even if model predictions are not entirely correct, the insights gained can be used to adjust, refine, or alter the model or a previously posed hypothesis, for instance, by accounting for mechanistic features suggested by experimental data. In this alternation of experimental and computational methods, one side informs and fertilizes the other, and iteratively both often improve.

As with any computational modeling strategy, it is important to note that clear goals and questions are needed up front, because they will determine the implementation details for the ABM. As a case in point, some early ABMs in biology lacked a unique objective and emphasized the replication of a biological phenomenon rather than the discovery of new characteristics or behaviors of a system. An early mesendoderm migration model, for instance, was framed around the functional incorporation of various elements into a model. These rather vague objectives can in retrospect be attributed to the fact that ABMs were relatively new in the field and that it was necessary to gain experience with exploring specific model features and the role of synergism among processes and rules. Since these early days of ABMs in biology, the field has substantially matured, and most modern ABMs are crisply focused on specific questions surrounding a particular application, as they should be.

One of the primary advantages of designing models for a specific application is that it becomes possible to simplify extraneous features. For example, in the first two crypt models, phenotypes were generalized and associated with location in the crypt rather than intrinsic fate decisions. This strategy was beneficial because the inclusion of phenotype would not have improved their analyses but would have added significantly more complexity.

Although the history of ABMs is quite short, trends suggest that these models are quickly becoming mainstream tools in biology. Not long ago, they simply tried to replicate what biologists were observing. In their next phase of development, they began to explain the roles of hypothesized mechanisms and their interactions. The field is now at the threshold of using ABMs for predictions of scenarios that had never been tested in the laboratory. Such predictions will not only help with the formulation of novel, testable hypotheses but may become a foundation for manipulating developing systems in a targeted manner, which will be fundamental to tissue engineering and regenerative medicine and possibly the creation of “tissue factories” that permit the production of pure, valuable organic compounds.
